# Flexible conducting polymer/reduced graphene oxide films: synthesis, characterization, and electrochemical performance

**DOI:** 10.1186/s11671-015-0932-1

**Published:** 2015-05-19

**Authors:** Wenyao Yang, Yuetao Zhao, Xin He, Yan Chen, Jianhua Xu, Shibin Li, Yajie Yang, Yadong Jiang

**Affiliations:** State Key Laboratory of Electronic Thin Films and Integrated Devices, School of Optoelectronic Information, University of Electronic Science and Technology of China (UESTC), Chengdu, 610054 People’s Republic of China

**Keywords:** Flexible, PEDOT-PSS, Reduced graphene oxide, Supercapacitor

## Abstract

In this paper, we demonstrate the preparation of a flexible poly (3,4-ethylenedioxythiophene) -poly (styrenesulfonate)/reduced graphene oxide (PEDOT-PSS/RGO) film with a layered structure via a simple vacuum filtered method as a high performance electrochemical electrode. The PEDOT-PSS/RGO films are characterized by scanning electron microscopy (SEM), X-ray diffraction, Raman spectroscopy, and Fourier transform infrared (FT-IR) spectrometry. The results indicate that a layer-ordered structure is constructed in this nanocomposite during the vacuum filtering process. The electrochemical performances of the flexible films are characterized by electrochemical impedance spectroscopy, cyclic voltammetry, and galvanostatic charge/discharge. The results reveal that a 193.7 F/g highly specific capacitance of nanocomposite film is achieved at a current density of 500 mA/g. This flexible and self-supporting nanocomposite film exhibits excellent cycling stability, and the capacity retention is 90.6 % after 1000 cycles, which shows promising application as high-performance electrode materials for flexible energy-storage devices.

## Background

Supercapacitors, also called electrochemical capacitors, are one of the important energy-storage devices with fast charging/discharging, high power density, high energy density, and long cycle life, which could fill the gap between the conventional capacitors and the batteries [1, 2]. Conductive polymers can store charges not only through pseudocapacitances but also in the electrical double-layer capacitances (EDLCs). The pseudocapacitance is due to faradic charge transfer, while the EDLCs could store energy by an ion adsorption-desorption process in the double electrical layer at the electrode/electrolyte interfaces [3, 4]. As a result, the conductive polymer electrodes present higher specific capacitance than that of pure carbon-based capacitors with EDLCs [5]. One of the most significant problems is that pure conducting polymers are usually mechanically weak or brittle, leading to poor cycling stability during the long cycle charge/discharge process [6, 7]. Coupling conductive polymers to a carbon material has been proven to be an effective approach to overcome this problem [8, 9]. The reduced graphene oxide (RGO), which exhibits high performance of electrical and electrochemical activity combined with favorable mechanical strength, can be obtained by reducing graphene oxide (GO) through chemical treatment [10, 11]. The nanocomposites, based on conducting polymer and RGO, exhibit chemical stability and ideal capacitive behavior when they are used as electrode materials [12–14].

Due to the fast development of flexible electronics, the requirement for high-performance flexible energy-storage devices is bursting. Furthermore, a self-supporting and collector-free supercapacitor electrode with large energy density and great mechanical strength is an important component for a flexible energy-storage device. Recently, much attention has been focused on the conducting polymer poly (3,4-ethylenedioxythiophene)-poly (styrenesulfonate) (PEDOT-PSS), which was used as a flexible electrode due to high conductivity and flexible processability [15–17].

In this paper, we demonstrate the preparation of flexible and self-supporting PEDOT-PSS/RGO films as an electrochemical electrode via a simple vacuum filtration method. In the first step, the GO was prepared by a modified Hummer’s method, and the RGO was obtained by a following hydrazine reduction treatment. Then, the PEDOT-PSS/RGO nanocomposites were prepared by an in situ solution polymerization. The PEDOT-PSS was polymerized on the surfaces of RGO sheets, and a sandwich-like PEDOT-PSS/RGO structure was formed. After that, the self-supporting nanocomposite films with a layer-by-layer structure were prepared by vacuum- filtered method. The PEDOT-PSS/RGO nanocomposite films showed better electrochemical properties compared with the pure RGO and PEDOT-PSS films. The combination of flexibility and electrochemical activity makes this self-supporting nanocomposite film attractive for applications in organic and flexible electrode materials.

## Methods

### Materials

Three, 4-ethylenedioxythiophene (EDOT) was purchased from Bayer Company (Leverkusen, Germany). Graphite flakes (Grade 3061) and Polystyrenesulfonate (PSS 25 %) were bought from Sigma-Aldrich (St. Louis., MO, USA). Cation exchange resin (001 × 7) and anion exchange resin (201 × 7) were purchased from Bohong Company (Tianjin, China). Ammonium persulphate ((NH4)_2_S_2_O_8_, 98 %), Mohr’s salt ((NH_4_)_2_Fe(SO_4_)_2_ · 6H_2_O, 99 %) and ethanol (C_2_H_6_O, 99.5 %) were purchased from Kelong Chemicals (Chengdu, China).

### Preparation of PEDOT-PSS/RGO self-supporting films

The RGO was synthesized from graphite flakes by the modified Hummer’s method and hydrazine reduction method [18, 19]. Deionized water and ethanol-mixed solution (volume ratio 1:1) was used to improve the solubility of EDOT. 100 mg RGO was dispersed in 200-mL mixture solution by sonicating for at least 2 h. In situ polymerization solution of PEDOT-PSS/RGO was prepared by introducing 1.4 mmol PSS and 0.7 mmol EDOT into the 200-mL RGO dispersion. This in situ polymerization solution was vigorously stirred and kept at 5 °C. Then, 1.4 mmol ammonium persulphate and 0.0014 mmol Mohr’s salt dissolved in 100 mL deionized water were added slowly while stirring overnight. Finally, the inorganic impurities in the final PEDOT-PSS/RGO dispersion were removed by 001 × 7 cation-exchange resin and 201 × 7 anion- exchange resin.

The PEDOT-PSS/RGO dispersion was sonicated for 2 h and vacuum filtered by PTFE filter membranes (pore size 0.22 μm, diam. 47 mm). Then the films were washed with ethanol and dried at 60 °C for 24 h in vacuum. After that, the dried films were stabilized at 235 °C in vacuum for 30 min and then peeled off from the filter to form self-supporting films.

The PEDOT-PSS/RGO self-supporting films synthesized from different mass ratios were labeled as PS/RG ratio. The PS/RG 1:1 indicates that the mass ratio of EDOT and RGO was 1:1. Meanwhile, the pure PEDOT-PSS and pure RGO self-supporting films were synthesized by a similar synthesis method mentioned above.

### Characterization and electrochemical measurements

The morphologies of PEDOT-PSS/RGO films were investigated with S-4800 scanning electron microscopy (SEM). X-ray diffraction (XRD) patterns were performed on an X’Pert PRO diffractometer with Cu Ka radiation (λ = 0.154 nm). Raman spectra were characterized with a Horiba LabRAM HR system (633 nm laser). Fourier transform infrared (FT-IR) spectra were characterized with a ThermoElectron Nicolet 6700 spectrophotometer. A CHI660D electrochemistry workstation was used to characterize the electrochemical performance of the samples at ambient temperature. Electrochemical impedance spectroscopy (EIS), cyclic voltammetry (CV), and galvanostatic charge/discharge (GCD) were performed with a 1-mol/L Na_2_SO_4_ aqueous electrolyte. The platinum foils and an Ag/AgCl electrode were used as counter and reference electrodes, respectively. All the measurements were performed at ambient temperature.

## Results and discussion

**Fig. 1:**
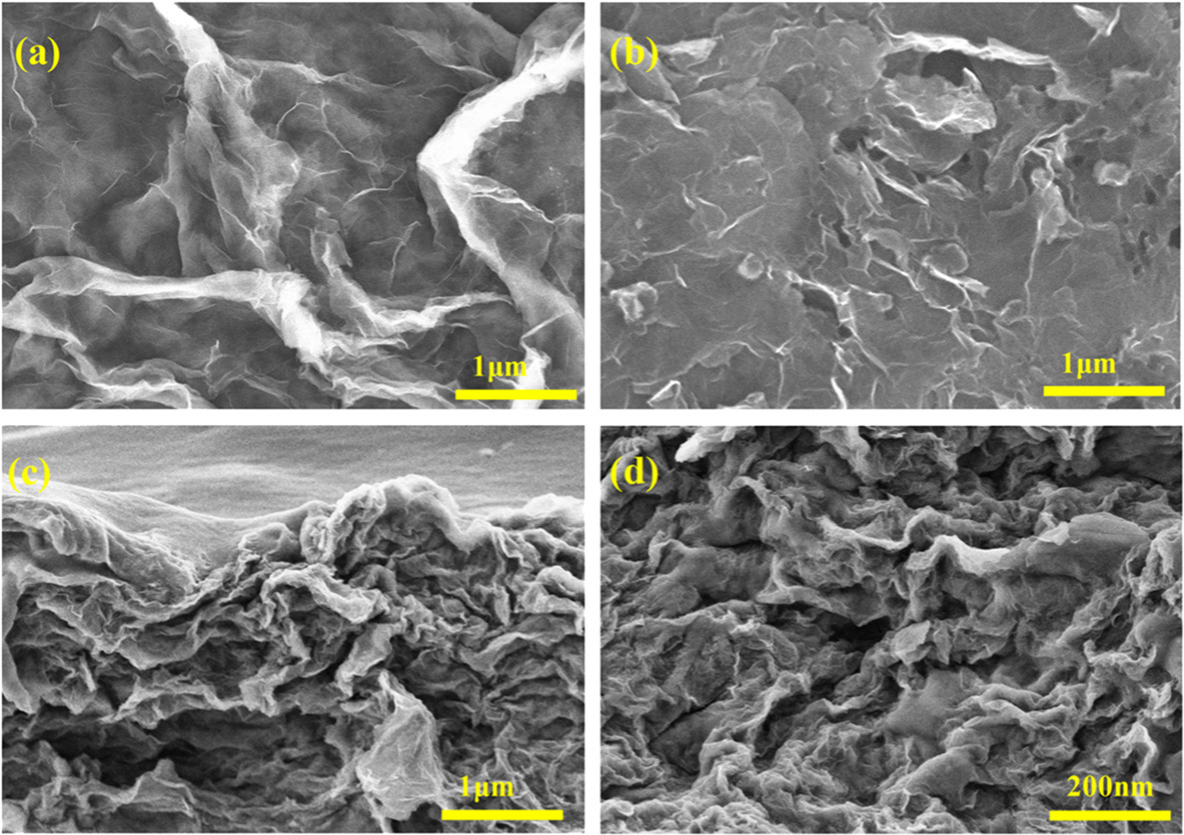
SEM images of flexible films as follows: (**a**) pure RGO, (**b**) PS/RG 1:1 and (**c**, **d**) cross-section images of PS/RG 1:1

The SEM images of pure RGO and PEDOT-PSS/RGO nanocomposite films are shown in Fig. 1. The changes of the microstructures of the films provide important information to reveal the strengthening mechanism of the nanocomposite films. The SEM image in Fig. 1a indicates that the pure RGO film is composed of a large quantity of curved nanosheets. As PEDOT-PSS/RGO nanocomposite films (shown in Fig. 1b), a porous 3D network structure with a few folds is presented. It is clear that the RGO nanosheets are homogeneously coated by PEDOT-PSS, indicating that PEDOT-PSS was successfully polymerized on the surfaces of RGO nanosheets to form sandwich-like structures. The cross-section of PEDOT-PSS/RGO nanocomposite film (shown in Fig. 1c and d) present a layer-by-layer formation, which is probably caused by the flow-assembly effect of RGO sheets during filtration [20–22].

**Fig. 2:**
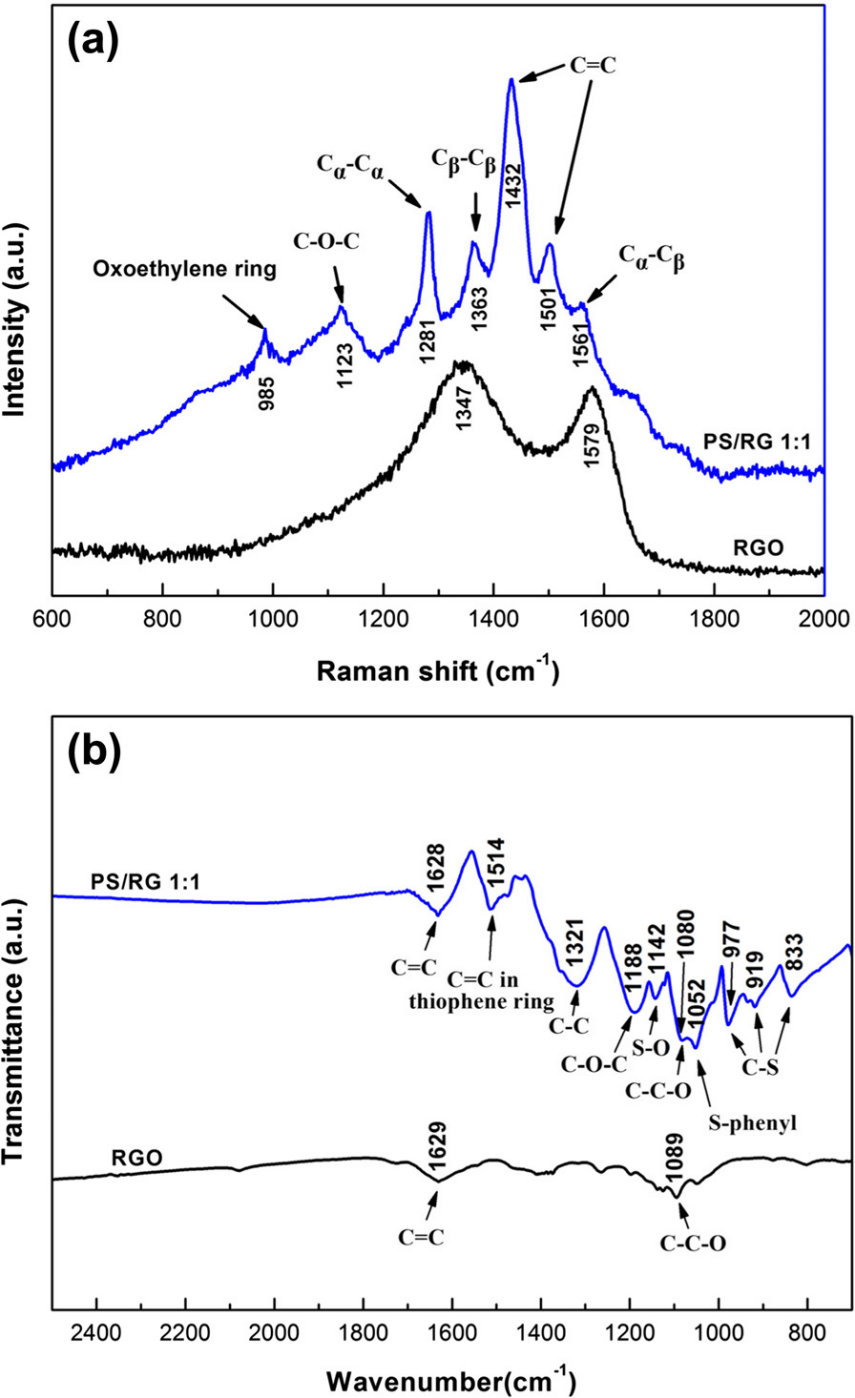
(**a**) Raman spectra of pure RGO and PS/RG 1:1 films; (**b**) FT-IR spectra of RGO, and PS/RG 1:1 films

Raman and FT-IR spectroscopy provide powerful tools to further investigate the microstructure of composites. Fig. 2a shows the Raman spectra of pure RGO and PEDOT-PSS/RGO composite films. The Raman spectrum of RGO displays two typical bands centered at 1347 and 1579 cm^−1^, corresponding to the D-band and G-band, respectively. The D-band indicates the increasing of the sp^3^ domains and the decrease of the in-plane sp^2^ domains. The G-band, due to the E_2g_ mode, is closely related to the vibration of sp^2^-bonded carbon atoms in a 2D graphene layer [23, 24]. The Raman band of PEDOT-PSS/RGO composite films (shown in Fig. 2b) at 985 cm^−1^ is assigned to oxyethylene ring deformation. The band at 1123 cm^−1^ is originated from C-O-C deformation. The bands at 1281 and 1363 cm^−1^ are originated from the C_α_-C_α_ inter-ring and C_β_-C_β_ stretch, respectively. The bands at 1432 and 1501 cm^−1^ are attributed to C = C symmetric stretch. The asymmetric C_α_-C_β_ bond is evidenced by the presence of band at 1561 cm^−1^ [23, 25]. The series of bands indicates the successful formation of PEDOT-PSS/RGO nanocomposites.

Figure 2b presents the FT-IR spectra of pure RGO and PS/RG 1:1 composite films. The spectrum of RGO film displays two peaks about at 1629 and 1089 cm^−1^, attributing to the aromatic C = C and oxygenated bond C-C-O, respectively [26]. We conclude that the oxygenated bond C-C-O arises from residual oxygen containing function groups in the RGO sheets, which improves the solubility of RGO. From the FT-IR spectrum of PS/RG 1:1, the spectrum contains peaks of C = C, C-C and C-O-C bonds in the thiophene ring at 1514, 1321, and 1188 cm^−1^ [8]. The C-S bond in the thiophene ring is evidenced by the presence of bands at about 977, 919 and 833 cm^−1^ [27]. Additionally, the bands at 1142 and 1052 cm^−1^ are attributed to the S-O bond and S-phenyl bond in PSS [28]. Moreover, the above-mentioned characteristic peaks of RGO are reflected in the spectrum of the PS/RG 1:1 film. However, the peak corresponding to the aromatic C = C and oxygenated bonds C-C-O are blue-shifted to 1628 and 1080 cm^−1^, suggesting interactions between PEDOT-PSS backbone and RGO sheets [29]. Therefore, the series of bands in FT-IR spectra indicates the successful formation of PEDOT-PSS coatings on the surfaces of the RGO.

**Fig. 3:**
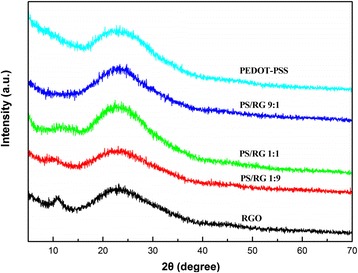
XRD patterns of RGO, PEDOT-PSS, and composite films

Figure 3 shows the XRD patterns of pure PEDOT-PSS, pure RGO, and PEDOT-PSS/RGO nanocomposite films with different RGO contents. The broad peak of PEDOT-PSS pattern ranging from 16.4 to 30.9 ° is attributed to PEDOT-PSS [30]. The pure RGO, PS/RG 1:9, and PS/RG 1:1 exhibits a small peak centered at 10.9 °. We conclude that this small peak arises from residual oxygen containing function groups in the RGO sheets, which improves the solubility of RGO. However, this small peak disappears in PS/RG 9:1 probably due to the low mass ratio of RGO. Compared with pure RGO, the XRD pattern of the PEDOT-PSS/RGO nanocomposites exhibits a broad peak ranging from 15 to 30 °, which is similar with RGO. The intensity of the peaks increases a little, which is ascribed to the reflection of PEDOT-PSS. Therefore, the XRD pattern further confirms the formation of PEDOT-PSS coatings on the surfaces of the RGO, which is consistent with that of the Raman investigations [31].

**Fig. 4:**
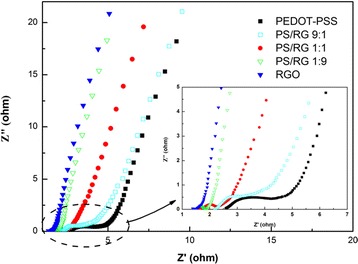
Nyquist plots of varied films. Inset shows plots with enlarged scale

The electrochemical performances of PEDOT-PSS/RGO films were analyzed by using EIS, CV, GCD, and cycle-life tests. The Nyquist plots of pure PEDOT-PSS, pure RGO, and PEDOT-PSS/RGO nanocomposite films with different RGO contents are showed in Fig. 4. It can be seen that all the plots exhibit the capacitive-type behaviors. It is well known that the first interception of the plots at the Z’ axis represents the equivalent series resistance (ESR), and the diameter of the semicircle is ascribed to the charge transfer resistance at the electrode/electrolyte interface [26]. As shown in Fig. 4, the ESR of the PEDOT-PSS, PS/RG 9:1, PS/RG 1:1, PS/RG 1:9, and RGO films are 2.56, 2.15, 1.62, 1.87, and 1.35 Ω, respectively. For the pure PEDOT-PSS and PS/RG 9:1 films, the radii of the semicircles in the Nyquist plot are larger than PS/RG 1:1, displaying a small semicircle characterization in high frequency range. This result indicates that the charge transfer resistance of PS/RG 1:1 film was lower than that of pure PEDOT-PSS and PS/RG 9:1 films. This might be due to the fact that the agglomeration of PEDOT-PSS films during the charge/discharge process decreased the effective surface area between the electrolyte and electrodes. Meanwhile, the inconspicuous semicircles at high frequency of PS/GR 1:9 and RGO films suggest that the interfacial charge transfer resistance is significantly low due to the high electrical conductivity of RGO [10]. The sloped portion closed to 45 ° in these plots at low frequency is typical of Warburg resistance, which is the result of the frequency dependence of ion transport or diffusion in the electrolyte [32, 33]. The Warburg region of RGO, PS/RG1:9, and PS/RG1:1 films are smaller than that of PEDOT-PSS or PS/RG 9:1 films, indicating that the shorter ion diffusion paths are formed during the charge/discharge process. Hence, we deduce that the more contents of RGO in nanocompoistes, the lower Warburg resistance is achieved in electrode materials. At the low frequency, all plots exhibit almost vertical straight lines, especially those films with a high mass ratio of RGO, indicating that these nanocomposite films exhibited an ideal capacitive behavior during the energy storage process. This result also reveals that a low-resistance interface was formed between the PEDOT-PSS and RGO, which is suitable for fast adsorption and desorption of solution ions.

**Fig. 5:**
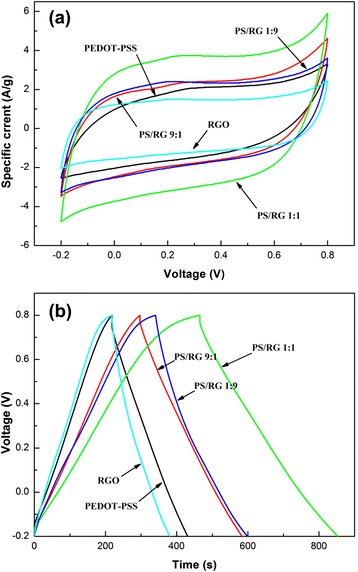
(**a**) Cyclic voltammograms curves of varied films at a scanning speed of 20 mV/s; (**b**) Galvanostatic charge to discharge curves of varied films at a current density of 500 mA/g

Figure 5a shows the CV curves of composite films within potential range from −0.2 to 0.8 V in a 1-M Na_2_SO_4_ electrolyte at a scan rate of 20 mV/s. It can be seen that the CV curves of all the films displayed an almost rectangular shape, indicating the good capacitive properties of RGO and PEDOT-PSS nanocomposites. It is clear that PEDOT-PSS/RGO films exhibit larger CV area than that of pure PEDOT-PSS and pure RGO films, revealing higher charge-storage capability of nanocomposites. It is well known that as an electrochemical capacitor electrode, the PEDOT-PSS/RGO nanocomposite films can afford both pseudo capacitance and EDLCs during the electrochemical energy storage process. The enhanced-charge storage capability is attributed to the well dispersion of RGO in PEDOT-PSS, providing a three-dimensional net work for charge transmission. The highly specific surface area of RGO and high pseudo capacitance of conducting polymer lead to excellent capacitance performance of nanocomposite electrode. As shown in Fig. 5a, the PS/RG 1:1 film shows the largest CV area corresponding to the highest specific capacitance, indicating the optimum contents of conducting polymer in nanocomposites. Accordingly, the decreasing of conducting polymer contents in nanocomposites causes lesser contribution of pseudo capacitance, leading to smaller CV area and specific capacitance. It needs to be mentioned that the lower contents of RGO in a composite film will not hinder the conductive polymer from agglomerating effectively during the charge/discharge process, leading to smaller specific capacitance. It has been found that a mass ratio of EDOT to RGO, about 1:1, was the optimum proportion to prepare PEDOT-PSS/RGO nanocomposites with excellent capacity performance. Moreover, the Fig. 5a also suggests that the PEDOT-PSS/RGO film exhibits an excellent electrical synergistic effect, and low contact resistance is formed between electrolyte and electrodes.

A comparison of capacitive performance was performed using galvanostatic charge/discharge curves at a constant current density of 500 mA/g, which is shown in Fig. 5b. It is clearly seen that the shape of the charge/discharge curves of varied films are closely triangular in shape, indicating a good reversibility during the charge/discharge processes. The discharge times of the PEDOT-PSS/RGO nanocomposite films are longer than that of the pure PEDOT-PSS and pure RGO films, suggesting the coating of the PEDOT-PSS on the RGO surface greatly extending the discharge time. Moreover, the PS/RG 1:1 film shows the longest charge/discharge time, indicating the largest specific capacitance of these electrode films. The specific capacitance can be calculated from the capacitance equation:

$$ {C}_m=\frac{i\varDelta t}{m\varDelta V} $$

Where the *C*_*m*_, *i*, *Δt*, *ΔV* and *m* represent the specific capacitance, discharge current, discharge time, discharge potential window, and mass of nanocomposite films, respectively. According to the capacitance equation evaluated from the slopes of the discharge curves, the specific capacitance of pure PEDOT-PSS, PS/RG 9:1, PS/RG 1:1, PS/RG 1:9, and pure RGO films are 106.3, 143.4, 193.7, 129.2, and 81.8 F/g, respectively. The highly specific capacitance in PEDOT-PSS/RGO films results from the synergetic effect between PEDOT-PSS and RGO, which was constructed with a layer-by-layer and porous structure. This special structure also results in improving electrolyte-ion transfer efficiency and better frequency performance of electrode films during the charge/discharge process.

**Fig. 6:**
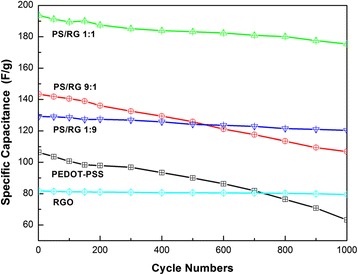
Cycling performances of varied films at a current density of 500 mA/g

The stability of different films as energy storage electrodes was evaluated by charge/discharge cycling tests at a current density of 500 mA/g (Fig. 6). The pristine specific capacitance of RGO is 81.8 F/g, and decreases slightly to 79.5 F/g (97.2 % retention) after 1000 cycles. The specific capacitance loss of RGO film is small, and the carbon nanomaterial shows excellent capacity-retention capability. In contrast, the PEDOT-PSS film shows higher specific-capacitance loss than RGO film with poor cycling performance. The capacitance value of PEDOT-PSS film decreases from 106.3 to 98.4 F/g in the first 150 cycles (7.4 % loss) and decreases slightly to 96.8 F/g during the next 150 cycles (1.5 % loss). An obvious decrease of specific capacitance is observed after the 1000 cycles (40.5 % loss), which is attributed to the poor mechanical capability of conducting polymer during the charge/discharge process, especially at high current density. The specific capacitance of PS/RG 1:1 film can maintained 90.6 % of initial capacitance (175.4 F/g) after 1000 cycles. Obviously, the addition of RGO into PEDOT-PSS improves the mechanical performance of PEDOT-PSS, which results in a better cycling stability of conducting polymer. The PS/RG 9:1 and PS/RG 1:9 films also present better capacity retention performance than PEDOT-PSS film and keep 74.5 % (106.8 F/g) and 93.1 % (120.3 F/g) of initial specific capacitance over 1000 cycles, respectively. It can be estimated that, under the proper mass ratio of RGO, more RGO in nanocomposites results in better cyclic stability of composite films. This result reveals that the RGO sheets provide a robust mechanical support for PEDOT-PSS, preventing conductive polymer from swelling and shrinking during the long-time charge/discharge process. Therefore, it has been found that an optimum proportion of EDOT to RGO with 1:1 is better to prepare composite electrode with excellent cycling performance, which is consistent with that of CV curves.

## Conclusions

We demonstrated preparation of flexible and self-supporting PEDOT-PSS/RGO nanocomposite films with a layered structure through a simple vacuum-filtered method. The superior capacitive performance of the PEDOT-PSS/RGO nanocomposites was confirmed by the tests of electrochemical properties. The mass ratio of PS/RG showed distinct influence on electrochemical performance of as-prepared composite electrodes. A 193.7 F/g highly specific capacitance was achieved at a current density of 500 mA/g. Furthermore, compared with pure PEDOT-PSS film, this nanocomposite film exhibited better energy-storage stability and can keep 90.6 % of the original specific capacitance after 1000 cycles.
